# Accurate and Robust Train Localization: Fusing Degeneracy-Aware LiDAR-Inertial Odometry and Visual Landmark Correction

**DOI:** 10.3390/s25154637

**Published:** 2025-07-26

**Authors:** Lin Yue, Peng Wang, Jinchao Mu, Chen Cai, Dingyi Wang, Hao Ren

**Affiliations:** 1 Signal and Communication Research Institute, China Academy of Railway Sciences, Beijing 100081, China; 2 School of Astronautics, Harbin Institute of Technology, Harbin 150001, China; 3 School of Electrical Engineering and Automation, Harbin Institute of Technology, Harbin 150001, China

**Keywords:** train positioning, degeneracy detection, LiDAR-inertial odometry, hierarchical visual detection

## Abstract

To overcome the limitations of current train positioning systems, including low positioning accuracy and heavy reliance on track transponders or GNSS signals, this paper proposes a novel LiDAR-inertial and visual landmark fusion framework. Firstly, an IMU preintegration factor considering the Earth’s rotation and a LiDAR-inertial odometry factor accounting for degenerate states are constructed to adapt to railway train operating environments. Subsequently, a lightweight network based on YOLO improvement is used for recognizing reflective kilometer posts, while PaddleOCR extracts numerical codes. High-precision vertex coordinates of kilometer posts are obtained by jointly using LiDAR point cloud and an image detection box. Next, a kilometer post factor is constructed, and multi-source information is optimized within a factor graph framework. Finally, onboard experiments conducted on real railway vehicles demonstrate high-precision landmark detection at 35 FPS with 94.8% average precision. The proposed method delivers robust positioning within 5 m RMSE accuracy for high-speed, long-distance train travel, establishing a novel framework for intelligent railway development.

## 1. Introduction

High-precision and long-duration train positioning serves as the foundation for safe train operation and information scheduling. In areas with unstable GNSS signals, such as the Qinghai–Tibet Railway, the currently prevalent train positioning solution involves a combination of wheel speed odometry and track transponders. However, this solution is flawed by issues such as low positioning accuracy and high construction and maintenance costs. With the advancement of artificial intelligence technologies, the railway transportation sector urgently needs to develop an intelligent train positioning solution [1,2,3].

Multi-sensor fusion schemes, combining cameras, LiDAR, IMU, and other sensors have gradually been applied in robots, unmanned aerial vehicles (UAVs), and autonomous vehicles [4]. Based on the coupling method, multi-sensor fusion schemes can be divided into loosely coupled and tightly coupled approaches. A representative work of the loosely coupled approach is LOAM, which separately processes LiDAR point cloud information and IMU inertial information to reduce the computational load. Simultaneously, LOAM uses IMU information as the initial system, but IMU measurement data are not involved in further optimization. The tightly coupled approach of LiDAR and IMU information is generally referred to as laser-inertial odometry. Laser-inertial odometry uses IMU information for state prediction, while LiDAR point cloud adjusts these predictions. This tightly coupled LiDAR-inertial odometry represents a promising intelligent positioning solution. However, when applied to long-distance railway train positioning over extended periods, it faces the following challenges: (a) Railway trains often traverse tunnels, bridges, forests, and other environments where GNSS signals are absent. (b) Loop closure detection does not occur during railway train travel, so the third challenge is how to eliminate cumulative errors in the absence of GNSS.

Addressing the aforementioned issues, a novel multi-sensor fusion framework integrating degeneracy-aware LiDAR-inertial odometry with visual landmark correction is proposed. Firstly, for issue (a), a LiDAR-inertial odometry framework tailored for railway train operating environments. By constructing an IMU pre-integration factor that accounts for the Coriolis effect and an odometry factor designed for degenerate states, the framework significantly enhances positioning accuracy and robustness in complex railway environments. Notably, a non-heuristic threshold is employed for degeneracy detection, effectively addressing odometry degradation caused by sparse or repetitive environmental features in railway scenarios. For issue (b), a hierarchical visual detection method is designed, which first detects kilometer posts alongside the railway and then recognizes their numbers to obtain absolute position measurements, thereby correcting the cumulative errors of the laser-inertial odometry. Ultimately, by fusing laser point cloud information, environmental perception information, and IMU information, this method achieves high-precision, long-duration, and long-distance railway train positioning. The main contributions are summarized as follows:

- An accurate and robust positioning scheme tailored for railway trains has been designed. The railway train operating scenario spans a large scale, so the IMU preintegration factor incorporates the Earth’s rotation compensation. By combining chi-square testing and eigenvalues, a non-heuristic threshold is adopted for degeneracy detection, which determines the weight of the LiDAR-inertial odometry in the factor graph. This approach achieves a high-frequency and highly robust positioning output.
- We proposed a kilometer post-based error correction method. The process begins with hierarchical visual landmark detection: a lightweight neural network identifies kilometer posts and their numerical identifiers, which are then combined with point cloud data to acquire a precise position. Subsequently, kilometer post constraints are established to correct the pose.
- Extensive testing and experimentation have been conducted on the proposed method in real railway train operating environments. Experimental results demonstrate that the proposed method can achieve high-precision positioning under high-speed, long-distance railway train operations. This provides a new avenue for the development of intelligent railway trains.

The remainder of this paper is organized as follows: Section 2 critically reviews prior works in railway localization, highlighting methodological limitations in degeneracy handling and absolute position referencing. Section 3 presents the methodological framework, including mathematical formulations of the degeneracy-aware LiDAR-inertial odometry and kilometer post correction method. Section 4 validates the proposed system through large-scale field tests under real-world operational conditions. Section 5 synthesizes key findings and discusses implementation challenges. For clarity, Table 1 defines essential technical terms used throughout this study.

## 2. Related Work

In recent years, academic researchers and railway research institutions around the world have conducted thorough and comprehensive studies on train positioning methods. In this section, we will present advanced research schemes in the areas of train section positioning and globally consistent train positioning. 

### 2.1. Train Section Localization

Train localization and scheduling are typically conducted through section-based localization, and most accurate train positioning information in railway systems worldwide originates from Train Control Systems (TCSs) [5]. This system comprises physically laid balises along the railway tracks and wheel speed sensors, where the balises provide accurate positions and the sensors estimate the distance between two balises. However, due to wheel slip and slide issues, this method exhibits low positioning accuracy in practical applications, and the installation of balises is costly and difficult to maintain. To address this issue, Global Navigation Satellite Systems (GNSSs) have been extensively researched and applied, fused with track odometers [6], wheel odometers [7], and Inertial Measurement Units (IMU) [8] to meet the real-time localization requirements of trains.

However, most of these methods overlook the potential assistance of environmental information in localization. In recent years, LiDAR-based or vision-based Simultaneous Localization and Mapping (SLAM) technology has been extensively researched and applied in autonomous vehicles, unmanned ships, aircraft, and other carriers [9,10,11,12]. On the one hand, to verify the feasibility of applying visual or visual-inertial SLAM in train localization, ref. [13] tested the performance of several advanced localization solutions such as VINS-mono and ORB-SLAM2 in railway localization. Experimental results indicate that vision-based methods are sensitive to lighting changes and feature point extraction is not stable enough, leading to significant scale drift over extended train operations. On the other hand, LiDAR-based SLAM typically exhibits higher accuracy and robustness. RailSLAM [14], which estimates train states based on general Bayesian theory, is an early representative scheme applying LiDAR SLAM to train localization. Tyler Daoust et al. [15] combined a point cloud alignment algorithm based on RQE with a sliding window filter for dead reckoning to solve train localization problems, achieving robust low relative localization and absolute mapping errors. In recent years, high-resolution and high-precision 3D LiDAR has been widely used in large outdoor scene localization. Yusheng Wang et al. [16] proposed the first real-time large-scale train SLAM solution, RailLoMer-V. This solution integrates LiDAR, IMU, wheel odometers, and GNSS based on a sliding window factor graph. Extensive experiments have demonstrated its high localization accuracy. However, this system requires a GNSS to provide absolute positions to eliminate cumulative errors, making it challenging to cope with tunnel segments where satellite positioning signals are denied for extended periods. To overcome this issue, ref. [17] fused LiDAR and IMU measurements in a tightly coupled manner and leveraged geometric constraints from common tunnel features such as electricity poles to address the degradation problem in tunnels. However, without absolute position constraints, this method can also accumulate significant errors or even fail after prolonged and long-distance operations.

### 2.2. Train Global Localization

Train operations are characterized by long durations and distances, and the localization errors of both LiDAR-based and vision-based SLAM methods will gradually accumulate over time [18]. Therefore, it is necessary to eliminate the accumulated errors at the end of each train operation section or within shorter distances to achieve globally consistent train localization. Currently, common methods include introducing GNSS signals, loop closure correction, and adding beacons along the railway. Mountainous railways that traverse tunnels and valleys often experience significant GNSS signal loss, and trains generally operate unidirectionally, making loop closure correction impractical. UWB beacons [19], optical beacons, image beacons, etc., which are uniquely tied to locations, are commonly used for localization correction. QR codes, barcodes [20], and specially designed tags [21] have proven effective in robot pose correction and landmark recognition [22]. Therefore, the detection and localization of landmarks are particularly important. Studies [23,24,25] achieved excellent results by using Monte Carlo simulation or neural networks to augment point cloud data before performing object extraction from the point cloud. However, it is difficult to obtain detailed semantic information on small landmarks from point clouds alone. Therefore, visual detection is a more ideal solution. Among visual target detection methods, the YOLO series algorithms [26,27,28], which balance efficiency and accuracy as a one-stage detector, have become among the most influential algorithms.

Many researchers have explored the application of visual landmarks in train localization. Ref. [29] fused LiDAR and IMU measurements for section localization, using visual landmarks along the railway to correct the pose estimates of the train’s laser-inertial odometer. D. Teng et al. [30] proposed a train image localization technique for scenarios with weak satellite signals to assist in train localization and correction. Image recognition technology is used to extract numbers on cooperative beacons, and the recognition results are transmitted to the train control system for matching with a prior map to generate train localization correction information, achieving accurate train localization.

## 3. Methodology

### 3.1. Problem Definition

**Figure 1:**
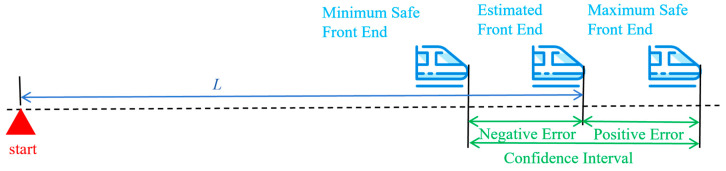
Train positioning schematic diagram.

Railway train positioning is a one-dimensional position estimation problem, aiming to determine the travel distance of the train’s front end along the railway line, the train speed, and the position confidence interval. Since the estimated travel distance of the train inevitably contains errors, this error range is called the confidence interval. It is determined by both the measurement accuracy of the sensors and the positioning algorithm. Taking the estimated train position as the nominal value and adding the confidence interval, the maximum safe front end and the minimum safe front end of the train are obtained, as shown in Figure 1.

The train positioning states required by TCS include estimated travel distance of the train’s front end, train speed, and positioning confidence interval.

(1) $$X={\left[L,v,\Delta L\right]}^{T}$$

Due to factors such as low cost and convenience, traditional train positioning solutions rely on a wheel speed sensor. To fully leverage the multidimensional information provided by advanced sensors such as LiDAR and IMU, we estimate the three-dimensional position and attitude information of the train, which is then further processed into the one-dimensional data required by the TCS. Therefore, as shown in Equation (2), the system state includes the system’s position, attitude, velocity, gyroscope bias, accelerometer bias, and gravity vector.

(2) $$x={\left[p,R,v,b_{g},b_{a},g\right]}^{T}$$

### 3.2. System Overview

**Figure 2:**
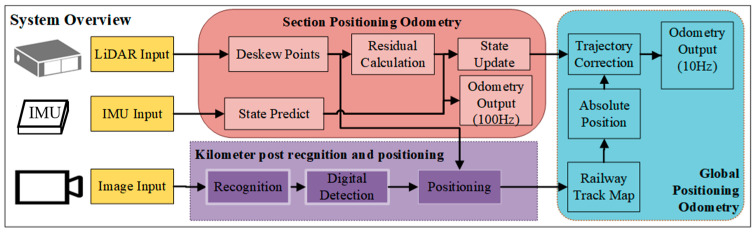
System overview.

The overall process of the proposed method is illustrated in Figure 2, which comprises three modules. The section positioning odometry is responsible for outputting the real-time motion pose during train travel. The hierarchical visual detector module is tasked with detecting the absolute position measurement information of kilometer posts. The global positioning odometry is dedicated to optimizing and correcting the errors.

**Figure 3:**
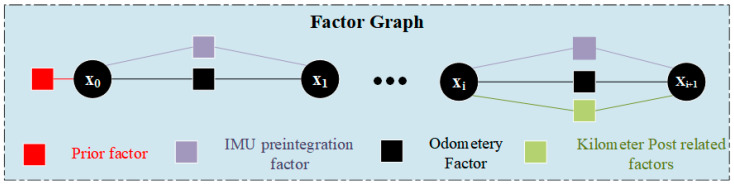
Factor graph optimization architecture diagram.

The train localization problem is modeled as a maximum a posteriori (MAP) estimation problem given the sensor measurements. As shown in Figure 3, this optimization problem is formulated on a factor graph relying on iSAM2 [31]. To ensure the real-time performance of the system, a parallel thread is constructed. Although graph-based optimization methods have been widely applied in SLAM systems, the size of the graph can grow significantly over time, leading to time-consuming computations. To manage the optimization process, the system only optimizes the poses of keyframes of point clouds. Keyframes are selected by setting appropriate thresholds based on temporal differences and spatial position deviations. In this paper, keyframe selection employs a temporal threshold of 1.0 s and a spatial threshold of 5.0 m relative to the last keyframe.

The optimal state of the system is obtained by minimizing the sum of the squared norms of each factor, as shown in Equation (3).

(3) $$x^{*}=\underset{x}{\arg\min}\{e_{𝒫}^{T}\Omega_{𝒫}e_{𝒫}+e_{ℑ}^{T}\Omega_{ℑ}e_{ℑ}+e_{𝒪}^{T}\Omega_{𝒪}e_{𝒪}+e_{𝒦}^{T}\Omega_{𝒦}e_{𝒦}\}\;$$

where $e_{𝒫}$ represents the prior factor marginalized through the Schur complement method; $e_{ℑ}$ denotes the IMU pre-integration residual; $e_{𝒪}$ denotes the odometry factor; and $e_{𝒦}$ denotes the residual between the kilometer post vertex and its corresponding vertex in the railway track map. Ω is the information matrix of the corresponding factor. The information matrix of the odometry factor and IMU preintegration factor are constructed based on the covariance matrices estimated by the ESKF or preintegration.

### 3.3. Section Positioning Odometry

The section positioning odometry is responsible for providing high-frequency estimates of the train’s pose through a lightweight and fast factor graph. This odometry incorporates both the IMU pre-integration factor and the odometry factor.

#### 3.3.1. IMU Preintegration Factor

Unlike typical SLAM systems, our IMU preintegration factor retains the Coriolis acceleration caused by the Earth’s rotation to enhance integration accuracy. SLAM is generally applied in small-scale, localized environments where the spatial extent is negligible compared to the scale of the Earth, and the vehicle speed is typically low. Thus, the effects of Earth’s rotation and Coriolis forces can often be disregarded. However, trains operate over long durations and distances, often traveling between provinces. Even the slowest passenger trains run at speeds of 120 km/h, while high-speed trains can reach 200–400 km/h. At these speeds, the impact of the Earth’s rotation must be carefully considered. Furthermore, the bias instability of consumer-grade IMU, tactical-grade IMU, and navigation-grade IMU is approximately 0.1–0.5 m/s^2^, 0.01–0.05 m/s^2^, and 0.001–0.005 m/s^2^, respectively. The Coriolis acceleration at a speed of 300 km/h is approximately 0.012 m/s^2^. SLAM systems commonly utilize low-cost commercial inertial measurement units, which typically exhibit measurement errors greater than those introduced by the Earth’s rotation or Coriolis effects. In our approach, we employ industrial-grade IMU (bias instability is 0.05 m/s^2^) to fully leverage the accuracy of the inertial navigation system. We eliminate negligible terms from the high-precision IMU model [32], retaining only the Coriolis acceleration due to the Earth’s rotation. The IMU motion model is as follows:

(4) $$\begin{array}{l} \dot{p}=v\\ \dot{v}=Rf+g-2[\Omega \times ]v\\ \dot{q}=\frac{1}{2}q⊗\left[\begin{array}{c} 0\\ \omega_{wb} \end{array}\right],\omega_{wb}=\omega_{ib}-R\Omega \end{array}$$

where ***f*** is the specific force and Ω represents the Earth’s rotational velocity.

Based on the IMU kinematic model in Equation (4) and referring to [33], we derive the IMU preintegration with Earth rotation compensation.

(5) $$\begin{array}{l} \Delta p={\left(R_{k-1}\right)}^{T}(p_{k}-p_{k-1}-v_{k-1}\Delta t-0.5g\Delta t^{2}+\Delta p_{cor})\\ \Delta v={\left(R_{k-1}\right)}^{T}(v_{k}-v_{k-1}-g\Delta t+\Delta v_{cor})\\ \Delta q={\left({\left(q_{k}\right)}^{-1}⊗q_{W_{i}(k-1)}⊗q_{k-1}\right)}^{-1} \end{array}$$

where subscripts *k* and *k* − 1 denote *t_k_* and *t_k−_*_1_, respectively, subscript *W_i_* denotes the historical world frame fixed to the IMU frame. The Coriolis corrections for both position and velocity are:

(6) $$\begin{array}{l} \Delta p_{cor}=2[\Omega \times ]\sum_{t_{m}}\left((p_{m}-p_{k-1})\Delta t_{m-1,m}\right)\\ \Delta v_{cor}=2[\Omega \times ](p_{k}-p_{k-1}) \end{array}$$

The IMU preintegration residual is defined as the difference between the estimated and measured increments of position, velocity, and orientation between two consecutive frames.

(7) $$e_{ℑ}=\left[\begin{array}{c} \Delta p-\alpha \\ \Delta v-\beta \\ 2[{\left(q_{k}\right)}^{-1}⊗q_{W_{i}(k-1)}⊗q_{k-1}⊗\gamma ] \end{array}\right]$$

where α,β,γ refer to the definitions in [34].

#### 3.3.2. Odometry Factor

The odometry factor is constructed from the system state obtained by the error state Kalman filter (ESKF), which jointly estimates IMU and LiDAR measurements [11]. The ESKF consists of two steps: prediction and update. The prediction process involves forecasting both the nominal state and the error state. The prediction of the nominal state is well established; here, we focus on handling the error state on SO(3). The dynamic equation of the system’s error state can be represented as follows:

(8) $$\delta \dot{x}=\left[\begin{array}{c} \delta v\\ -{\left(\omega_{m}-b_{g}\right)}^{\wedge }\delta \theta -\delta b_{g}-n_{g}\\ -R{\left(a_{m}-b_{a}\right)}^{\wedge }\delta \theta +R\delta b_{a}-Rn_{a}+\delta g\\ n_{bg}\\ n_{ba}\\ 0 \end{array}\right]$$

The original point cloud information is processed to remove motion distortion. Point-to-plane residuals are then constructed with local planes, and these residuals are used as the measurement model for updating. The measurement equation for the *j*-th LiDAR point cloud is shown in Equation (9):

(9) $$z_{j}=u_{j}^{T}(p_{j}^{W}-q^{W})$$

where $u_{j}$ is the normal vector of the local plane corresponding to point $p_{j}^{W}$ in the world coordinate system and $q^{W}$ is a point within the local plane corresponding to $p_{j}^{W}$.

Through the prediction-update process of the ESKF, we can obtain a relatively accurate estimate of the train’s current state, which allows us to construct the odometry factor:

(10) $$e_{𝒪}=\lambda t_{i-1}^{T}t_{i}$$

where λ is the normalized degradation factor and $t_{i-1}$ represents the position of the *i* – 1th keyframe of the laser point cloud.

It is noteworthy that we employed the non-heuristic adaptive degradation detection factor proposed in [35], which avoids the issue of manually setting detection factor thresholds as seen in [36]. The eigenvalues extracted from the information matrix in Equation (9) are normalized and arranged in ascending order as follows:

(11) $$\lambda ={\left(\lambda_{1},\lambda_{2},\lambda_{3}\right)}^{T}$$

The non-heuristic threshold for degradation detection is defined using the chi-squared test, where the null hypothesis states that each normalized eigenvalue follows its expected value. The chi-squared test formula for rejecting the null hypothesis is given by:

(12) $$\sum \frac{{\left(\lambda_{i}-e_{i}\right)}^{2}}{e_{i}}>0.103$$

where 0.103 represents the chi-squared value for 2 degrees of freedom at the 95% significance level.

According to the eigenvalue distribution of finite-dimensional real symmetric matrices, the expected values for each eigenvalue are 0.289, 0.498, and 0.749, resulting in the degradation detection thresholds as follows:

(13) $$\bar{\lambda }=e-\sqrt{0.103\times e}$$

The calculated degradation thresholds corresponding to the three eigenvalues are 0.12, 0.27, and 0.48. If any λ falls below its corresponding $\bar{\lambda }$, the odometry factor will no longer be included in the factor graph, thus primarily relying on the IMU preintegration factor and the kilometer post factor.

### 3.4. Hierarchical Visual Detector

The designed hierarchical visual detector is primarily responsible for detecting kilometer posts alongside the railway first, and then detecting the numbers on the kilometer posts. Subsequently, in the next section, kilometer post factors will be constructed to correct the cumulative errors of the LiDAR-inertial odometry.

#### 3.4.1. Detection of Kilometer Post

**Figure 4:**
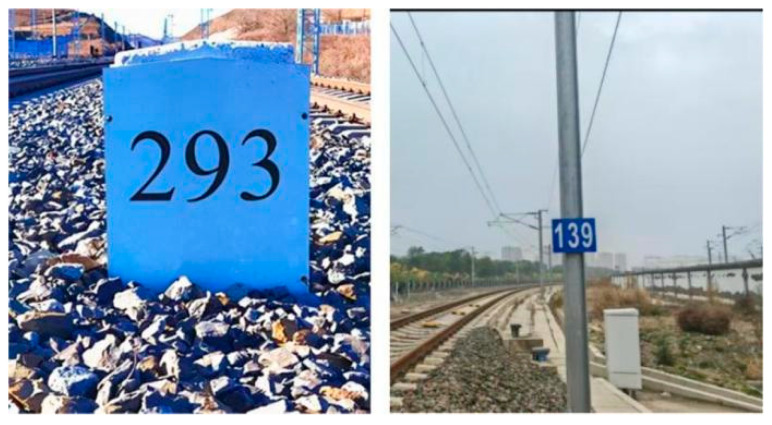
Comparison of two types of kilometer posts. Traditional stone post currently deployed along railways (**left**) and proposed lightweight kilometer post with enhanced reflectivity (**right**).

As shown in Figure 4, a new type of low-cost kilometer post alongside the railway has been designed to address the issues of existing stone kilometer posts being susceptible to wear and tear and lacking absolute numeric information. The surface of this kilometer post is attached with high-quality reflective film, which aids in the reflection of point cloud information by LiDAR. Additionally, the numeric values on the kilometer post alongside the railway carry explicit positional information, providing a foundation for subsequent measurements of the absolute pose.

Firstly, the region of interest (ROI) is input for the images captured by the system-mounted camera. This ROI corresponds to the area where kilometer posts are placed on both sides of the railway tracks, which has already undergone calibration and verification.

Subsequently, target detection is performed on the ROI of the captured images to detect the kilometer posts. We chose YOLOv5 for kilometer post detection because it maintains high accuracy while reducing the number of parameters, improving inference speed, and featuring a smaller model size suitable for deployment on embedded devices.

**Figure 5:**
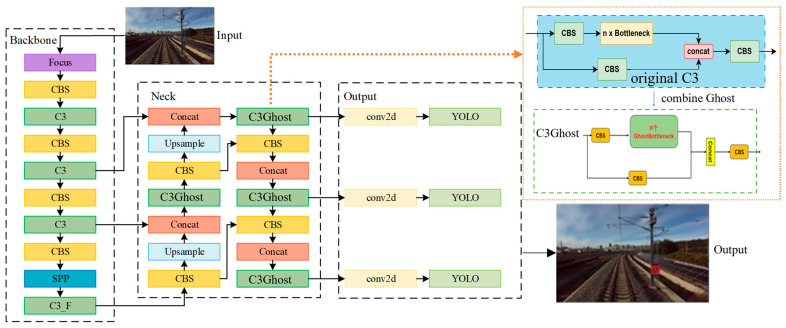
The model architecture of lightweight YOLOv5 for kilometer post detection.

However, as illustrated in Figure 5, the C3 module in YOLOv5 adopts CSPNet’s architecture to reduce computational redundancy by splitting input feature maps into two parallel streams: one retains identity through residual connections, while the other undergoes feature transformation via bottleneck modules before concatenation. To achieve lightweight convolution operations within the C3 module, we integrate the ghost convolution mechanism from GhostNet. It decouples intrinsic and cheap feature maps with the original C3’s efficient feature extraction capability. This fusion yields the proposed C3Ghost module, replacing the standard C3 blocks in the neck network. The complete kilometer post detection pipeline is detailed in Figure 5.

#### 3.4.2. Acquisition of Absolute Position

**Figure 6:**
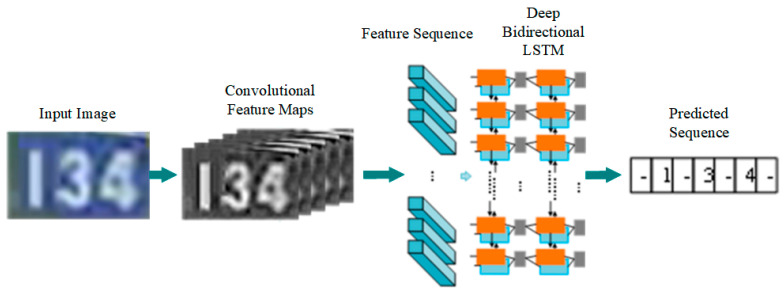
Schematic diagram of the PaddleOCR technical architecture for kilometer post number recognition.

After obtaining the coordinates of the bounding box region, the digital information within this region is recognized through the method of Optical Character Recognition (OCR). As shown in Figure 6, PaddleOCR technology has been selected for the detection of kilometer post numbers alongside the railway tracks. It is an OCR technology based on the deep learning framework PaddlePaddle. This technology boasts ultra-lightweight design, small model size, and ease of deployment on mobile and embedded systems.

**Figure 7:**
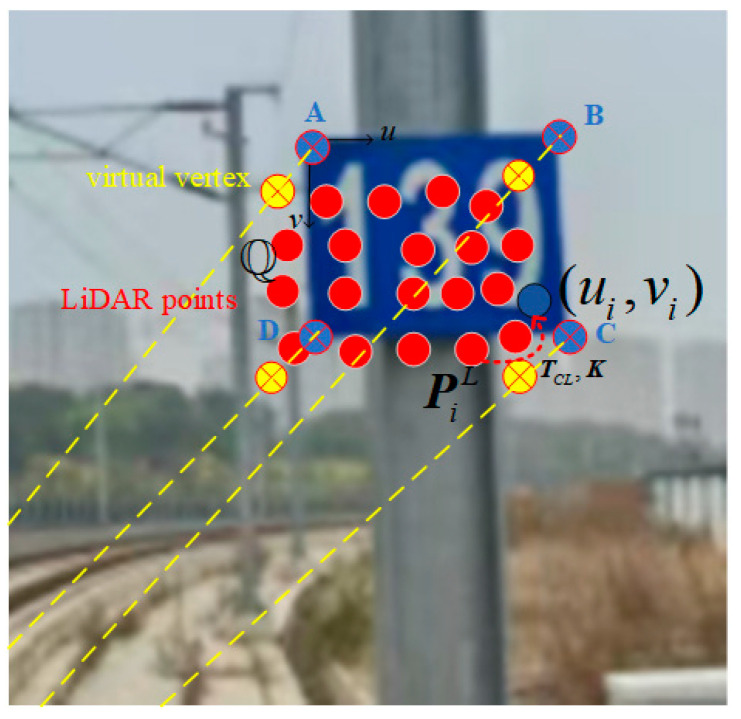
Schematic diagram of vertex coordinates of kilometer post. where A, B, C, and D are the actual kilometer post vertices, respectively, and the yellow dotted line is the hypothetical laser ray emitted from the LiDAR.

Within the bounding box area, after recognizing kilometer post numerals using PaddleOCR technology, the camera images are aligned with laser point clouds. The point cloud plane corresponding to the bounding box area is fitted, and combined with the frustum to obtain the vertex coordinates of the kilometer post. The specific process is as follows:

As shown in Figure 7, the kilometer posts are installed vertically on the side of the track, and the vertex coordinate of the bounding box of the kilometer post beside the track identified through object detection is denoted as $\left(u_{A},v_{A}\right)$, $\left(u_{B},v_{B}\right)$, $\left(u_{C},v_{C}\right)$, $\left(u_{D},v_{D}\right)$.

Assuming the laser point cloud at time *i*, and with the extrinsic parameter matrix between the LiDAR and camera pre-obtained through offline calibration, the laser point cloud is projected onto the camera coordinate system, as shown in Equation (14):

(14) $$P_{i}^{C}=R_{CL}P_{i}^{L}+t_{CL}$$

By combining the camera intrinsic parameter matrix with Equation (14), the pixel coordinates corresponding to the laser point cloud can be obtained, as shown in Equation (15):

(15) $$z_{i}^{C}\left[\begin{array}{c} u_{i}\\ v_{i}\\ 1 \end{array}\right]=\left[\begin{array}{ccc} f_{x} & 0 & c_{x}\\ 0 & f_{y} & c_{y}\\ 0 & 0 & 1 \end{array}\right]\left[\begin{array}{c} x_{i}^{C}\\ y_{i}^{C}\\ z_{i}^{C} \end{array}\right]=KP_{i}^{C}$$

For points that fall within the kilometer post bounding box region after projection, the RANSAC algorithm is utilized to fit and obtain the point cloud set of the kilometer post plane alongside the track. The equation of this plane can be expressed as:

(16) $$n_{a}x+n_{b}y+n_{c}z+n_{d}=0$$

Through inverse projection calculation, the coordinates $P_{A}^{L}$, $P_{B}^{L}$, $P_{C}^{L}$, $P_{D}^{L}$ of the four vertices of the kilometer post in the LiDAR frame can be obtained. Taking vertex A as an example, as shown in Equations (17) and (18), $P_{A}^{C}$ and $P_{A}^{L}$ can be solved.

(17) $$P_{A}^{C}=K^{-1}\left[\begin{array}{c} u_{A}\\ v_{A}\\ 1 \end{array}\right]z_{A}^{C}$$

(18) $$P_{A}^{L}=R_{CL}^{-1}(P_{A}^{C}-t_{CL})$$

Similarly, the coordinates of the other three vertices in the LiDAR frame can also be solved by combining Equations (16)–(18).

### 3.5. Global Positioning Odometry

During the operation of the system, each frame of the input point cloud is detected. If a kilometer post is detected by the hierarchical visual detector in the previous section and the absolute position information measurement of the kilometer post alongside the track is obtained, the system will incorporate it as a constraint term into the objective function of nonlinear optimization, constructing a large-scale and robust optimization problem to eliminate the cumulative error of the odometry and obtain a globally consistent trajectory and map.

Therefore, a kilometer post vertex factor is used to constrain the train’s position. Using the method described in Section 3.4, the coordinates of the 4-kilometer post vertices are obtained by combining the bounding box with the point cloud plane. The residual of the kilometer post vertex is defined as follows:

(19) $$\delta \theta =\sum_{l=1}^{4}Log(R_{P_{l}}^{T}(R_{WM}^{T}R_{M_{kl}}))$$

(20) $$\delta t=\sum_{l=1}^{4}(RP_{l}^{L}+t-M_{kl})$$

In the equation, δθ represents the rotational residual, δt represents the translational residual, $R_{WM}$ denotes the rotation from the train’s positioning world coordinate system to the electronic map coordinate system, and $M_{kl}$ represents the *l*-th vertex of the kth kilometer post in the electronic map.

The residual of the kilometer post vertices alongside the track can be divided into rotational residual and translational residual:

(21) $$e_{𝒦}={\left[\begin{array}{cc} \delta \theta & \delta t \end{array}\right]}^{T}$$

The information matrix is as follows:

(22) $$\Omega_{𝒦}=\left[\begin{array}{cc} \Omega_{\theta } & 0\\ 0 & \Omega_{t} \end{array}\right]$$

where $\Omega_{\theta }=\Sigma_{\theta }^{-1}$, with $\Sigma_{\theta }=\mathrm{diag}(0.0175^{2},0.0175^{2},0.0175^{2})$, and $\Omega_{t}=\Sigma_{t}^{-1}$, with $\Sigma_{t}=\mathrm{diag}(0.05^{2},0.05^{2},0.05^{2})$. The covariance values derive from experimental data. Using the kilometer post vertex positioning method described in Section 3.4.2, a typical angular error of 1° (i.e., 0.0175 radians) and a positioning error of 0.05 m are adopted.

The estimated train position, obtained through graph-based optimization, follows a Gaussian distribution.

(23) $$\hat{p}∼N(p,\sigma_{p})$$

The one-dimensional running distance of the train can be calculated using Equation (24):

(24) $$L=\sum_{i}{\left\|p_{i+1}-p_{i}\right\|}_{2}$$

Further, the confidence interval for the train’s position can be calculated from the corresponding covariance matrix. The 95% confidence interval is established as follows:

(25) $$\left(L-2\Delta_{L},L+2\Delta_{L}\right)$$

where $\Delta_{L}$ represents the norm of the standard deviation of the diagonal elements of $\sigma_{p}$.

## 4. Experiments

### 4.1. Experimental Setup

(1) Hardware setup

**Figure 8:**
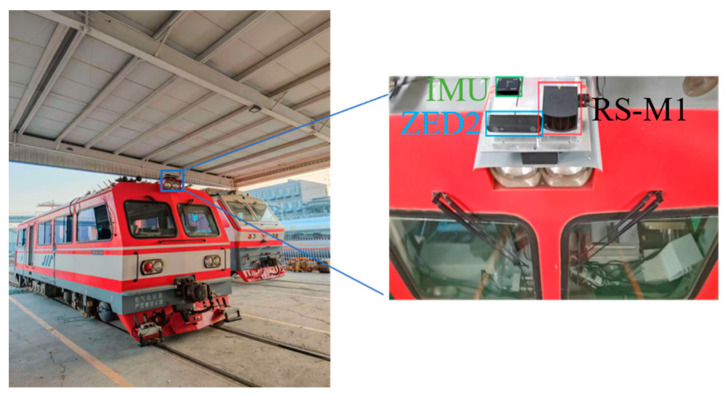
Experimental track vehicle and the experimental equipment on its top.

Our sensor equipment was mounted on a railway train, as shown in Figure 8. The equipment configuration is detailed in Table 2, which includes solid-state LiDAR, IMU, visual cameras, and GNSS. Notably, the GNSS information is only used as a reference truth value.

(2) Dataset details

We conducted data collection using the equipment shown in Figure 8 under real railway operational scenarios. As presented in Table 3, the train was operated along predefined routes at varying speeds during morning, noon, and evening sessions. Four representative data packages were selected for detailed analysis. These experimental sequences encompass complex characteristics including different operating speeds, lighting conditions, and travel distances, comprehensively replicating the diverse typical working conditions encountered in actual railway train operations.

(3) Evaluation metrics

In this paper, the root mean square error (ATE_RMSE_) and the maximum value (ATE_MAX_) of ATE [37] are adopted as evaluation metrics for localization accuracy. Their calculation methods are as follows:

(26) $${\mathrm{ATE}}_{\mathrm{RMSE}}=\sqrt{\frac{1}{N}\sum_{i=1}^{N}{\left\|t_{\mathrm{est},i}-t_{\mathrm{gt},i}\right\|}^{2}}$$

(27) $${\mathrm{ATE}}_{\mathrm{MAX}}=\max({\left\|t_{\mathrm{est},i}-t_{\mathrm{gt},i}\right\|}^{2}),i=1,\ldots ,N$$

where $t_{\mathrm{est},i}$ and $t_{\mathrm{gt},i}$ denote the estimated position and ground-truth position of the *i*-th frame, respectively. *N* represents the number of all coordinate points.

Additionally, since the train control system ultimately requires one-dimensional position information, the error between the estimated trajectory length and the true trajectory length (*L*_gt_) is defined as another important evaluation criterion:

(28) $$\mathrm{LE}=\left|L-L_{\mathrm{gt}}\right|$$

In the visual landmark recognition experiment, average precision (AP) was used as the primary evaluation metric, while frames per second (FPS) was used to quantify the model’s real-time capability.

### 4.2. Ablation Study

(1) Effect of Earth rotation compensation

In Table 4, we evaluated the effect of incorporating the Earth’s rotation compensation into IMU preintegration. High-precision inertial measurement units have gyroscope biases smaller than the Earth’s rotation rate, making it more critical to account for the Earth’s rotation compensation. The experimental results demonstrate that after compensating for the Earth’s rotation, the improved preintegration model achieves higher accuracy. Notably, the compensation yields marginal improvements in short-distance scenarios (e.g., morn_1_202411041117 and noon_3_202411061502). However, for high-precision positioning applications exceeding 10 min of operation or 20 km in distance—particularly during north–south trajectories—Earth rotation compensation demonstrates significant accuracy enhancement.

(2) Effect of non-heuristic degradation detection

**Figure 9:**
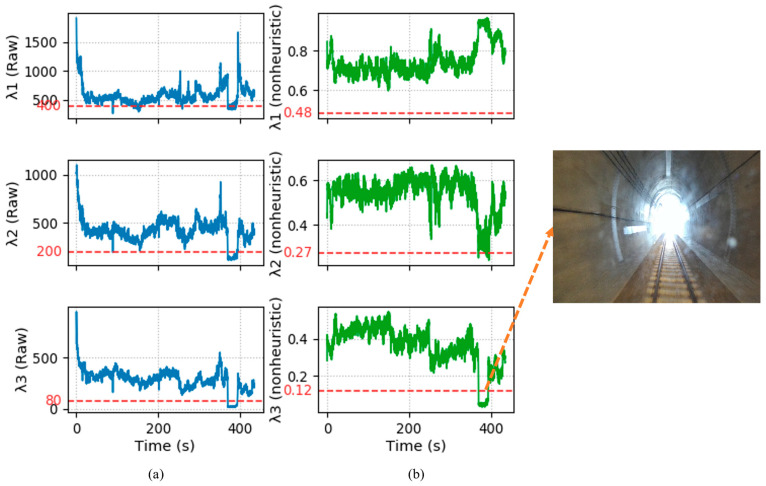
Degraded scene detection results. (**a**) Degradation factor curve of the method in reference [30], where the red dashed line threshold is manually set. (**b**) Degradation factor curve of the proposed method, where the red dashed line represents a fixed non-heuristic threshold.

We tested the effectiveness of the non-heuristic degeneracy detection method. As shown in Figure 9, the proposed approach does not require threshold adjustments and can effectively detect degraded scenes such as tunnels and bridges. As shown in Figure 9a, the method in [30] requires running the algorithm in advance to manually set an appropriate threshold based on the degradation factor curve and prior information. Moreover, unreasonable threshold settings can occur when it is impossible to pre-determine whether degradation happens along the corresponding direction, leading to misjudgment. For example, the three peaks around 100 s, 180 s, and 250 s in the figure are falsely identified as degradation scenarios. In contrast, the proposed method can effectively identify degradation scenarios such as tunnels and bridges without requiring manual threshold adjustment. As illustrated in Figure 9b, the tunnel area experiences severe degradation along the corresponding eigenvector direction (i.e., along the train’s forward direction), while only slight degradation occurs along the corresponding eigenvector direction (i.e., the rolling direction). No degradation occurs along the corresponding direction altogether, thereby avoiding the misjudgment problems inherent in traditional methods. Once degradation is detected, the weight of the LiDAR-inertial odometry is reduced, mitigating the impact of degradation and thereby achieving stable localization.

Furthermore, extensive testing was conducted in diverse scenarios including indoor corridors, open outdoor areas, and highway tunnels. Performance was evaluated using precision and recall metrics. Experimental results demonstrate that the method [30] achieved 91% precision and 92% recall. Our proposed method attained 96% precision and 99% recall representing improvements of 5% in precision and 7% in recall, respectively.

(3) Effect of kilometer post factor

**Figure 10:**
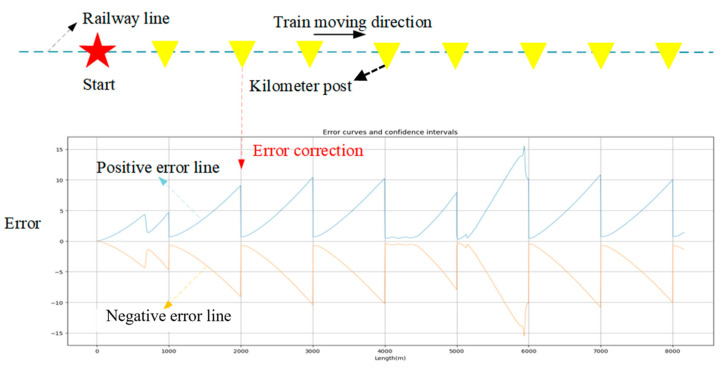
Confidence interval variation curve of train positioning.

To verify the error elimination effect of the kilometer post factor, as shown in Figure 10, we plotted the curves of confidence interval changes, where the blue line represents the positive error curve, and the orange line represents the negative error curve.

As can be seen from Figure 10, as the train moves forward, the positioning error gradually accumulates, so the confidence interval gradually increases. The maximum confidence interval per kilometer is approximately 10 m. When a kilometer marker is detected, the global positioning odometry performs a global positioning correction, thereby eliminating the accumulated error and reducing the confidence interval back to near zero. Therefore, the kilometer post factor has an excellent effect on eliminating accumulated errors, ensuring accurate long-distance positioning of the train.

### 4.3. Hierarchical Visual Detector Experiments

Due to the lack of publicly available railway kilometer marker datasets, this paper independently constructed a kilometer marker image dataset. The data source was selected from front-view videos captured on an actual operational railway line, covering complex scenarios with varying day–night lighting conditions, rain and fog interference, and environments such as tunnels and bridges. After careful selection, a benchmark dataset containing 3089 high-resolution images was established. The LabelImg tool was used to annotate bounding boxes and class information for the kilometer posts.

To evaluate the performance of the lightweight kilometer marker detection model, experiments were conducted using the PyTorch 1.12.1 framework and Python 3.8 development environment, deployed on a workstation equipped with an NVIDIA GeForce RTX 3090Ti GPU (NVIDIA Corporation, Santa Clara, CA, USA) with 24 GB VRAM Performance evaluation comprehensively considered both detection accuracy and real-time performance.

Experimental results show that the original YOLOv5s achieves an AP of 95.2 and a frame rate of 150 FPS, while the proposed lightweight model achieves a slightly lower AP of 94.8 but reaches a significantly higher frame rate of 180 FPS. This demonstrates that the proposed model maintains high recognition accuracy while achieving faster detection speed, which is beneficial for deployment on embedded platforms.

**Figure 11:**
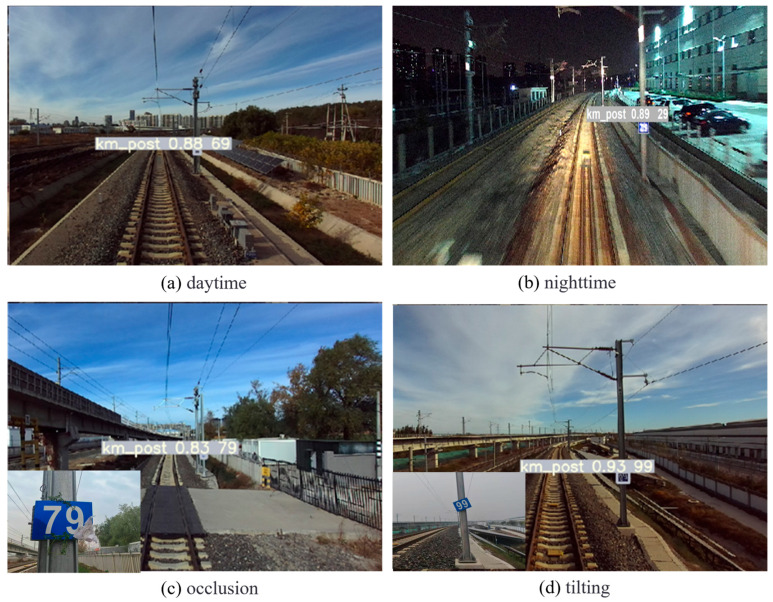
Kilometer post detection results in different test conditions.

In order to verify the application effectiveness of the kilometer post detection method based on the YOLOv5s model proposed in this paper in real-world environments, the newly designed kilometer posts were installed at regular intervals on the catenary poles along both sides of the track, approximately 2 m above the ground. Multiple repeated experiments were conducted during both daytime and nighttime, with the train traveling at speeds of 30 km/h, 60 km/h, and 90 km/h, respectively. The kilometer post recognition results are shown in Figure 11. The experimental results demonstrate that all kilometer posts can be accurately identified and their numeric information successfully extracted under different train speeds and different light conditions. It is worth noting that the model can still accurately identify kilometer posts even when they are tilted or partially occluded, demonstrating its strong generalization capability.

### 4.4. Accuracy Evaluation

This part evaluates the positioning performance of our system. The comparison methods include FAST-LIO2 [3], LIO-SAM [10], and LVI-SAM [38]. FAST-LIO2 is a fast, robust, and versatile LiDAR-inertial odometry framework built upon an efficient tightly coupled iterative Kalman filter. LIO-SAM is a LiDAR-inertial odometry system based on factor graph construction, which can integrate various data such as numerous relative measurements, GNSS, and loop closures as factors into the LiDAR-inertial odometry system. LVI-SAM proposes a tightly coupled LiDAR-visual-inertial odometry through smoothing and mapping. LVI-SAM is constructed on a factor graph and comprises two subsystems: a visual-inertial system (VIS) and a LiDAR-inertial system (LIS). These two subsystems are designed to be tightly coupled, where the VIS leverages estimates from the LIS to facilitate initialization. By comparing with these three advanced LiDAR odometry systems, the effectiveness and efficiency of the proposed method in long-distance and long-duration scenarios can be validated. It is important to note that during our testing, the loop closure detection function of all methods was disabled, as there are no loops in real railway train operating environments.

For noon_202411061502, the quantitative evaluation results are presented in Table 5. The proposed method outperforms the other three methods in terms of both APE and total trajectory length. This superiority is mainly attributed to the ability of the proposed method to identify kilometer posts alongside the railway tracks, which helps to suppress the divergence in system errors. Conversely, FAST-LIO2 and LIO-SAM cannot achieve this function, resulting in larger divergent errors. The LVI-SAM method experiences significant divergence, which is due to the fact that some parts of the trajectory sequence occur within tunnels and bridges, causing the LVI-SAM method to fail in localization.

However, in our method, due to the adoption of non-heuristic degradation detection, as shown in Figure 9, the proposed approach does not require threshold adjustments and can effectively detect degraded scenes such as tunnels and bridges. Once degradation is detected, the weight of the LiDAR-inertial odometry is reduced, mitigating the impact of degradation and thereby achieving stable localization.

**Figure 12:**
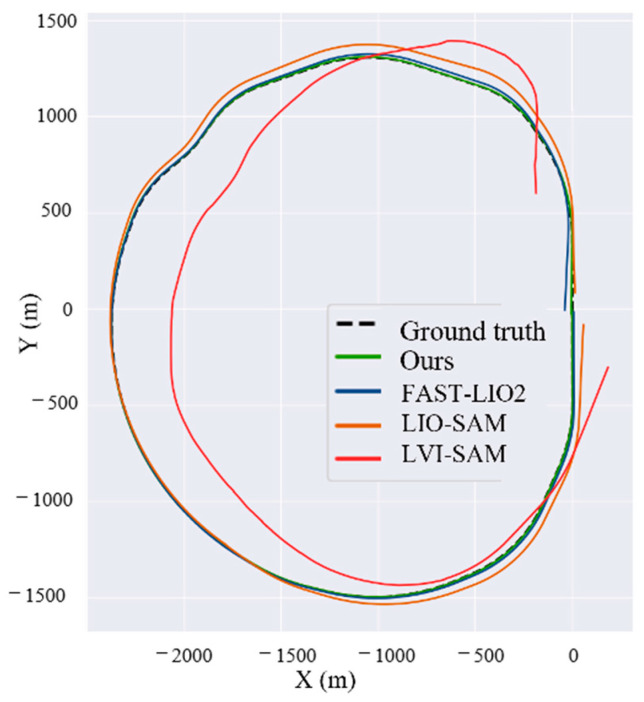
Trajectory of different methods.

Next, we qualitatively evaluated the trajectory errors of the test sequences, as shown in Figure 12. This qualitative result is consistent with the previous quantitative analysis. The proposed method achieved the most accurate positioning trajectory that closely matches the real-world scenario. The performance of FAST-LIO2 and LIO-SAM was weaker than the proposed method. The LVI-SAM method exhibited significant deviations from the true trajectory due to localization failures in tunnel environments.

In addition, for the other three datasets, we evaluated the ATE_RMSE, and LE of our method. In the sequences morn_202411041117, noon_202411061331, and night_202411061918, the LE values were 2.15 m, 8.18 m, and 14.83 m, respectively, while the ATE_RMSE_ remained below 5 m in all cases. This demonstrates that the proposed method can effectively handle various train operation scenarios with high accuracy and stability.

### 4.5. Time Analysis

The third part of the testing involves analyzing the implementation performance of the proposed algorithm. We tested the processing speed of the proposed method on an embedded system, as shown in Table 6. The section positioning odometry runtime is only 23 ms, which fully meets the requirement for high-precision, high-frequency pose information output in railway train positioning processes. Although global positioning odometry takes approximately 90 ms, it operates in a parallel thread and only optimizes the poses of all keyframes between two detected kilometer posts when a kilometer post is detected. Therefore, it has almost no impact on the real-time performance of the system. Additionally, the hierarchical visual detection module takes 28.57 ms, which guarantees multiple detections of kilometer posts alongside the railway tracks during high-speed train travel, enhancing measurement accuracy and robustness. At the same time, it is worth emphasizing that the hierarchical visual detection process runs in parallel with the odometry. And it employs GPU acceleration for detection, making the proposed algorithm an overall highly real-time intelligent positioning algorithm.

## 5. Conclusions

In this paper, we proposed an accurate and robust train localization method by fusing degeneracy-aware LiDAR-inertial odometry with visual landmark correction. This solution does not rely on GNSS and transponders, and it achieves higher accuracy compared to traditional train positioning methods while incurring lower deployment and maintenance costs. By introducing an IMU preintegration model that accounts for the Earth’s rotation, the system significantly improved long-distance dead reckoning accuracy, reducing positioning errors by about 30%. To address common degenerate scenarios, we developed an adaptive degeneracy detection mechanism based on chi-square testing, eliminating the subjectivity of manual parameter tuning. Additionally, by combining LiDAR-image joint localization with absolute position factor constraints, we achieved continuous high-precision positioning without GNSS signals or loop closure. Extensive datasets and real-world experiments demonstrate that the kilometer post recognition achieves an accuracy of 94.8% and can run in real time on embedded devices. Ablation studies show that the kilometer post factor plays a significant role in position correction. Across four representative datasets, the system exhibited robust performance and real-time capability, with ATE_RMSE consistently below 5 m.

Despite these advancements, challenges remain, particularly in handling extreme weather conditions and further improving computational efficiency for real-time applications. Future research will focus on enhancing the system’s resilience under adverse environmental conditions, further improving the accuracy of kilometer post recognition, and utilizing characteristics specific to railway environments to further constrain the train’s pose. The ultimate goal is to achieve comprehensive coverage of train operations across all speed ranges, scenarios, and time periods. These findings provide a solid technical foundation for the development of intelligent railways.

## Figures and Tables

**Table 1:** Definitions and explanations of variables.

Variable Names	Variable Contents
x	States related to train positioning
δx	The difference between the true state and the nominal state denoted as Error State
${\left(\cdot \right)}^{L},{\left(\cdot \right)}^{I},{\left(\cdot \right)}^{C},{\left(\cdot \right)}^{W}$	LiDAR Frame, IMU Frame, Camera Frame, World Frame defined by IMU initial frame
$T_{AB},R_{AB},t_{AB}$	Extrinsic parameters between coordinate system A and coordinate system B
Log(·)	Logarithmic map from SO(3) to Euclidean space

**Table 2:** Configuration of experimental equipment.

Equipment	Description
RS-M1 solid-state LiDAR (RoboSense Technology Co., Ltd., Shenzhen, China)	Irregular scanning pattern, FoV: 120° × 25°, 10 Hz
Nvidia jetson agx orin (NVIDIA Corporation, Santa Clara, CA, USA)	12-core Arm Cortex-A78AE 64-bit CPU, 64 GB RAM
G90 integrated navigation suite (WHEELTECH Co., Ltd., Dongguan, China)	Contains a 9-axis IMU (8°/h(1σ) ) and GNSS positioning receiver, used to provide ground truth trajectories
ZED2 camera (Stereolabs, San Francisco, CA, USA)	20 Hz, 960 × 540 resolution, only the left camera is used for image capture

**Table 3:** Dataset details.

Dataset	Distance (m)	Duration (s)
morn_202411041117	3910.54	470
noon_202411061502	8375.53	1195
noon_202411061331	19,020.51	1263
night_202411061918	24,103.19	1120

**Table 4:** Trajectory error evaluation results.

Dataset	LE (w/o ERP *)	LE (w/ERP)
morn_202411041117	16.85	14.11
noon_202411061502	26.87	22.08
noon_202411061331	106.48	78.64
night_202411061918	125.34	86.21

* represents Earth rotation compensation.

**Table 5:** Quantitative results of positioning errors.

Method	ATE_MAX_ (m)	ATE_RMSE_ (m)	Length Error (m)
FAST-LIO2	343.91	165.86	18.77
LIO-SAM	342.13	234.39	42.46
LVI-SAM	738.26	1125.91	1187.19
Ours	9.89	3.88	3.74

**Table 6:** Analysis of temporal performance.

Settings	Module	Time (ms)
LIDAR: 10 Hz	Section positioning odometry	22.99
Camera: 20 Hz, 960 × 540	Global positioning odometry	89.54
IMU: 100 Hz	Hierarchical visual detector	28.57

## Data Availability

Restrictions apply to the availability of the data. Data can be obtained from the first author.
